# The number of risk factors not at target is associated with cardiovascular risk in a type 2 diabetic population with albuminuria in primary cardiovascular prevention. Post-hoc analysis of the NID-2 trial

**DOI:** 10.1186/s12933-022-01674-7

**Published:** 2022-11-07

**Authors:** Ferdinando Carlo Sasso, Vittorio Simeon, Raffaele Galiero, Alfredo Caturano, Luca De Nicola, Paolo Chiodini, Luca Rinaldi, Teresa Salvatore, Miriam Lettieri, Riccardo Nevola, Celestino Sardu, Giovanni Docimo, Giuseppe Loffredo, Raffaele Marfella, Luigi Elio Adinolfi, Roberto Minutolo, NID-2 study group Investigators, U Amelia, C Acierno, P Calatola, O Carbonara, G Conte, G Corigliano, M Corigliano, R D’Urso, A De Matteo, L De Nicola, N De Rosa, E Del Vecchio, G Di Giovanni, A Gatti, S Gentile, L Gesuè, L Improta, A LampitellaJr, A Lampitella, A Lanzilli, N Lascar, S Masi, P Mattei, V Mastrilli, P Memoli, R Minutolo, R Nasti, A Pagano, M Pentangelo, E Pisa, E Rossi, F C Sasso, S Sorrentino, R Torella, R Troise, P Trucillo, A A Turco, S Turco, F Zibella, L Zirpoli

**Affiliations:** 1 Department of Advanced Medical and Surgical Sciences, University of Campania “Luigi Vanvitelli”, Piazza Luigi Miraglia 2, 80138 Naples, Italy; 2 Department of Physical and Mental Health and Preventive Medicine, Medical Statistics Unit, University of Campania “Luigi Vanvitelli”, Piazza Luigi Miraglia 2, 80138 Naples, Italy; 3 Department of Precision Medicine, University of Campania “Luigi Vanvitelli”, Via De Crecchio 7, 80138 Naples, Italy; 4 Division of Cardiovascular Sciences, Faculty of Biology, Medicine and Health, The University of Manchester, 3.31 Core Technology Facility, 46 Grafton Street, Manchester, M13 9NT UK

**Keywords:** Type 2 diabetes mellitus, Cardiovascular risk, Diabetes complications, Multifactorial treatment

## Abstract

**Background:**

Nephropathy in Diabetes type 2 (NID-2) study is an open-label cluster randomized clinical trial that demonstrated that multifactorial intensive treatment reduces Major Adverse Cardiac Events (MACEs) and overall mortality versus standard of care in type 2 diabetic subjects with albuminuria and no history of cardiovascular disease. Aim of the present post-hoc analysis of NID- 2 study is to evaluate whether the number of risk factors on target associates with patient outcomes.

**Methods:**

Intervention phase lasted four years and subsequent follow up for survival lasted 10 years. To the aim of this post-hoc analysis, the whole population has been divided into 3 risk groups: 0–1 risk factor (absent/low); 2–3 risk factors (intermediate); 4 risk factors (high). Primary endpoint was a composite of fatal and non-fatal MACEs, the secondary endpoint was all-cause death at the end of the follow-up phase.

**Results:**

Absent/low risk group included 166 patients (52.4%), intermediate risk group 128 (40.4%) and high-risk group 23 (7.3%). Cox model showed a significant higher risk of MACE and death in the high-risk group after adjustment for confounding variables, including treatment arm (HR 1.91, 95% CI 1.04–3.52, P = 0.038 and 1.96, 95%CI 1.02–3.8, P = 0,045, respectively, vs absent/low risk group).

**Conclusions:**

This post-hoc analysis of the NID-2 trial indicates that the increase in the number of risk factors at target correlates with better cardiovascular-free survival in patients with type 2 diabetes at high CV risk.

**Clinical Trial Registration:**

ClinicalTrials.gov number, NCT00535925. https://clinicaltrials.gov/ct2/show/NCT00535925

**Supplementary Information:**

The online version contains supplementary material available at 10.1186/s12933-022-01674-7.

## Background

Diabetes confers about a two-fold excess risk of coronary heart disease, stroke, and death due to other vascular causes, as shown in a large meta-analysis from over 100 prospective studies [1]. Moreover, it has been known for approximately 30 years that independent of the number of major cardiovascular (CV) risk factors, diabetes per se increases the risk of CV death two to three times compared to non-diabetics [2].

Therefore, the reduction of CV morbidity and mortality in diabetic patients, with the consequent direct and indirect cost savings, is one of the main challenges of health systems around the world.

The clinical management of diabetic patients becomes increasingly important in patients at higher CV risk and involves most diabetic patients from the diagnosis of the disease.

The recent ESC/EASD guidelines state that the diabetic patient may have moderate, high or very high CV risk [3]. One of the most clinically relevant aspects of these guidelines is that they removed the distinction between primary and secondary CV prevention in high risk diabetic patients. Up to now, almost all randomized clinical trials (RCTs) distinguished patients based on whether they have already had a cardiovascular event or not. Instead, the ESC/EASD guidelines state that diabetic patients with multiple risk factors or even one organ damage belong to the same very high CV risk group as subjects on secondary prevention with established CV disease. Therefore, this new position of scientific societies certainly not only clarifies the clinical management of risk factors in diabetic patients, but could also modify the design, and in particular the selection of patients in future RCTs.

At present, the recent Nephropathy In Diabetes type 2 (NID-2) study, in type 2 diabetic subjects with albuminuria and diabetic retinopathy (DR) on primary CV prevention, has shown that multifactorial intensive treatment is able to reduce major adverse cardiovascular events (MACEs) and overall mortality [4]. Therefore, a diabetic population on primary CV prevention, but defined *ante litteram* at very high CV risk, has been shown to benefit from multifactorial intensive treatment for the first time in a multicenter RCT.

However, it is still unclear whether a gradual increase in the number of at target risk factors directly correlates to patient outcome. Therefore, the aim of the present post-hoc analysis of NID- 2 study is to evaluate the relationship between the number of risk factors that reached the predetermined threshold and CV outcome of patients.

## Methods

### Study design

This is a secondary analysis of the NID-2 study, an open-label cluster randomized clinical trial in patients with type 2 diabetes mellitus (T2DM) referred to 14 Italian diabetology clinics. The study design is detailed elsewhere [4]. Briefly, the centers have been randomly assigned to either multifactorial intensive therapy (MT) or Standard-of-Care (SoC). A questionnaire confirmed that all physicians of both arms were well aware of the guidelines on T2DM management published at the time of the study [5–8].

All MACE diagnoses have been made according to the diagnostic criteria defined by the international standard of care guidelines [9–11]. MACEs have been evaluated by cardiologists blinded to the study arm (MT or SoC), either belonging to the study centres or to other hospitals where patients were referred for acute events.

### Participants and procedures

T2DM patients with age ≥ 40 years, confirmed albuminuria ≥ 30 mg/24 h, severe DR, diabetes onset at age > 30 years, absence of neoplastic/psychiatric diseases and follow-up at the centre ≥ 12 months, were considered eligible [12]. Exclusion criteria were previous myocardial infarction (MI) or stroke, severe liver or heart failure.

As elsewhere described, 395 patients were randomized (207 to MT arm and 188 to SoC arm) between October 2005 and October 2008 [4]. The intervention phase was scheduled for a period of four years and was completed in December 2011. Patients were followed until May 2019 to measure the number of events required for the primary outcome.

In the SoC group, the subjects received the therapy usually administered at their diabetic clinic for the management of blood pressure, glycaemic and lipid control, and antiplatelet treatment, according to good clinical practice.

In the MT group, patients were treated with a pre-specified algorithm for the management of hypertension, glycaemic control and dyslipidaemia. This consists of implementing non-pharmacological and pharmacological treatments including physical activity, low sodium diet, renin–angiotensin system blockade (RAS), low-dose aspirin and statin [4].

The protocol has been approved by the ethics committee of University of Campania “Luigi Vanvitelli” (clinicaltrials.gov: NCT00535925) and is in accordance with the 1976 Declaration of Helsinki and its later amendments. All participants have signed their informed consent.

### Landmark analysis

A landmark analysis approach has been used to assess the effect of various risk factors on the endpoints of the study, mortality and a composite of fatal and non-fatal MACEs. Specifically, the landmark was set at the end of the treatment period of each individual patient with the assigned therapy in the main study. From that point, it has been assessed in the follow-up period until the occurrence of the death or composite MACE event.

### Variables

At the landmark, response to treatment and the achievement of the following targets: (a) systolic blood pressure (SBP) < 130 mmHg, (b) diastolic blood pressure (DBP) < 80 mmHg, (c) glycated hemoglobin (HbA1c) < 7%, (d) fasting serum low density lipoprotein (LDL) cholesterol < 100 mg/dL were assessed. Failure to meet the targets has been assessed as the presence of a risk factor. Based on the number of risk factors, the whole population was divided into 3 risk groups: 0–1 risk factor (absent/low); 2–3 risk factors (intermediate); 4 risk factors (high).

The primary endpoint was a composite of fatal and non-fatal MACEs, including CV mortality, non-fatal MI (documented instrumentally and/or enzymatically), non- fatal stroke, coronary artery bypass, revascularization procedures (PTCA) and lower limb major amputation, whichever occurred first. The secondary endpoint was all-cause death at the end of the follow-up phase.

### Statistical analysis

Categorical data have been expressed as numbers and percentages, while continuous variables have been presented as either median and interquartile range or mean and standard deviation, based on their distribution assessed by the Shapiro–Wilk test. Differences in each variable and comparison of more than two group were evaluated using ANOVA procedure or Kruskal–Wallis test, respectively for continuous variables with normal or skewed distribution, or Pearson’s chi-squared for categorical data. Median follow-up time has been calculated by the inverse Kaplan–Meier procedure. Cox regression model with shared-frailty has been used to calculate hazard ratio (HR) and 95% confidence interval (CI). In the univariate analysis, only presence of risk factors has been included as covariate. Multivariable analyses have been adjusted by age and treatment received at randomization. Furthermore, the interaction between risk groups and treatment arm was also tested within this model. We performed three sensitivity analyses: first, we assessed the role of missing data by comparing patients with absent information for at least one of the variables included in the score (missing group) with the rest of the patients (non-missing group); second, we evaluated different grouping of the risk factors by grouping risk factors as 0–1, 2 and 3–4; third, we combined high SBP and/or DBP in one risk factor. Proportionality assumption was checked using log–log plot of survival and tested using Schoenfeld residuals. The Number Needed to Treat (NNT) has been calculated using the hazard ratios and the survival probability in the control group, as proposed by Altman [13]. Data have been analyzed using STATA 16.0 software (StataCorp. 2019. College Station, TX: StataCorp LLC).

## Results

Out of the 395 enrolled and randomized patients (207 to MT arm and 188 to SoC arm), 368 were evaluated at the landmark time for the present analysis. The median durations of the intervention phases were 3.84 and 3.40 years in the MT and SoC groups respectively. 51 patients (30 to MT and 21 to SoC arm) had absent information for at least one of the variables included in the score and were classified as missing.

As depicted in Table 1, the absent/low risk group included 166 patients (52.4%, 62 with 0 and 104 with 1 risk factor), the intermediate risk group 128 patients (40.4%, 82 with 2 and 46 with 3 risk-factors) and the high-risk group (4 risk factors) 23 patients (7.3%). Patients' characteristics at landmark analysis overall and by risk group are reported in Table 2. As expected, more patients from MT arm were stratified in the absent/low risk group while patients originally randomized in the SoC arm predominated in the intermediate and high-risk groups. No major intergroup difference emerged in terms of age, sex, kidney function and hemoglobin.

**Table 1:** Distribution of risk factors in the three risk groups

	Risk groups				
	Absent/low		Intermediate		High
Number of risk factors	0	1	2	3	4
Patients (number)	62	104	82	46	23
Type of risk factor					
SBP ≥ 130 mmHg (n, %)	0 (0.0%)	12 (11.5%)	30 (36.6%)	39 (84.8%)	23 (100.0%)
DBP ≥ 80 mmHg (n, %)	0 (0.0%)	6 (5.8%)	14 (17.1%)	25 (54.3%)	23 (100.0%)
HbA1c ≥ 7% (n, %)	0 (0.0%)	22 (21.2%)	54 (65.9%)	34 (73.9%)	23 (100.0%)
LDL ≥ 100 mg/dL (n, %)	0 (0.0%)	64 (61.5%)	66 (80.5%)	40 (87.0%)	23 (100.0%)

*SBP* systolic blood pressure, *DBP* diastolic blood pressure

**Table 2:** Overall patient’s characteristics at the end of interventional phase

	Overall	Groups			P
		Absent/low risk	Intermediate risk	High risk	
N	368	166	128	23	
Age (years)	70.4 ± 9.2	69.7 ± 9.8	71.2 ± 8.2	70.0 ± 11.7	0.43
Males (n, %)	173 (47%)	80 (42.8%)	54 (42.2%)	12 (52.2%)	0.49
SBP (mmHg)	130.9 ± 12.8	125.4 ± 7.3	135.3 ± 13.7	148.3 ± 11.5	< 0.001
DBP (mmHg)	78.4 ± 7.3	76.5 ± 5.2	79.2 ± 7.8	91.1 ± 5.0	< 0.001
Serum creatinine (mg/dL)	1.2 ± 0.6	1.2 ± 0.6	1.3 ± 0.7	1.3 ± 0.4	0.13
eGFR (mL/min/1.73m^2^)	60.5 ± 22.7	63.3 ± 22.4	56.7 ± 22.9	56.4 ± 19.3	0.033
Albuminuria (mg/day)	80 [26–180]	100 [30–190]	60 [18–150]	94 [60–230]	0.033
Hemoglobin (g/dL)	12.9 ± 1.6	12.9 ± 1.5	12.6 ± 1.8	13.2 ± 1.5	0.13
Glycaemia (mg/dL)	150.2 ± 42.0	139.4 ± 27.6	157.9 ± 42.9	169.4 ± 59.4	< 0.001
HbA1c (%)	7.2 ± 0.9	6.8 ± 0.6	7.4 ± 0.9	8.1 ± 0.8	< 0.001
Total cholesterol (mg/dL)	181.1 ± 32.1	166.6 ± 24.5	193.8 ± 30.6	222.0 ± 24.7	< 0.001
HDL-cholesterol (mg/dL)	44.4 ± 12.8	43.9 ± 9.8	44.9 ± 15.9	41.7 ± 7.2	0.50
LDL-cholesterol (mg/dL)	110.4 ± 30.1	98.3 ± 25.9	119.7 ± 27.6	147.6 ± 21.5	< 0.001
Triglycerides (mg/dL)	143.4 ± 58.1	135.2 ± 50.0	146.3 ± 59.7	163.3 ± 42.5	0.030
Therapeutic goals					
SBP < 130 mmHg (n, %)	234 (67.2%)	154 (92.8%)	59 (46.1%)	0 (0.0%)	–
DBP < 80 mmHg (n, %)	276 (79.3%)	160 (96.4%)	89 (69.5%)	0 (0.0%)	–
HbA1c < 7% (n, %)	203 (56.9%)	144 (86.7%)	40 (31.3%)	0 (0.0%)	–
LDL < 100 mg/dL (n, %)	131 (39.6%)	102 (61.4%)	22 (17.2%)	0 (0.0%)	–
Treatment arm*					< 0.001
MT	199 (54.1%)	116 (58.3%)	51 (25.6%)	2 (1.0%)	
SoC	169 (45.9%)	50 (29.6%)	77 (45.6%)	21 (12.4%)	

Data are mean ± SD or median and [IQR]. Comparisons are evaluated using ANOVA procedure or Kruskal–Wallis test, respectively for continuous variables with normal or skewed distribution, or Pearson’s chi-squared for categorical data

*SBP* systolic blood pressure, *DBP* diastolic blood pressure, *eGFR* glomerular filtration rate estimated by CKD-EPI formula, *MT* multifactorial intensive treatment, *SoC* standard of care

*distribution of different risk groups among the study arms is presented as row relative frequency

**Fig. 1:**
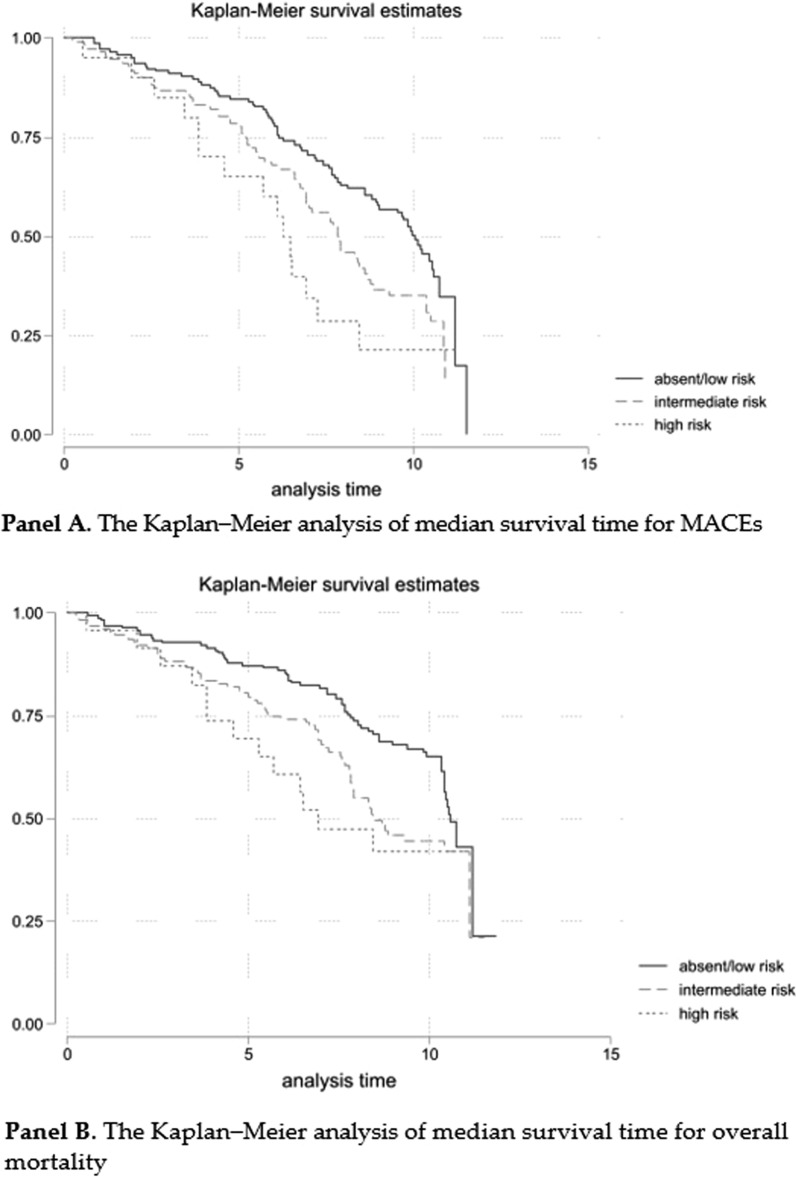
Kaplan–Meier analysis of median survival time for MACEs (Panel A) and overall mortality (Panel B)

During follow-up (median 10.0 years, 95%CI 9.9–10.3) 188 MACEs and 162 deaths were recorded. The Kaplan–Meier analysis shows a median survival time for MACEs of 10 years (95% CI 8.9–10.6) in the 0–1 risk factor group, 7.9 years (95% CI 6.9–8.7) in the 2–3 risk factor group and 6.3 years (95% CI 3.8–7.3) in the 4-risk factor group (Fig. 1, Panel A). Evaluating overall mortality, the Kaplan–Meier curve shows a median survival time of 10.6 years (95% CI 10.4–*NA*) in the 0–1 risk factor group, 8.4 years (95% CI 7.8–*NA*) in the 2–3 risk factor group and 6.9 years (95% CI 4.6–*NA*) in the 4-risk factor group (Fig. 1, Panel B). The Cox models, using the absent/low group as reference, showed significant differences at univariate model and after adjustment for confounding variables (Table 3). In the adjusted models treatment effect was not significant. Furthermore, the interaction between the risk groups and the treatment arm was not statistically significant. The assumption of proportionality was not violated by any of the independent variables in the model for both MACE and overall survival (OS) outcome (Additional file 1: Tables S1 and S2).

**Table 3:** Risks for MACEs and all-cause mortality in the three risk groups at univariate Cox model and after adjustment for confounding variables

	MACEs						All-cause mortality					
	Model 1			Model 2			Model 1			Model 2		
	HR	95%CI	p	HR	95%CI	p	HR	95%CI	p	HR	95%CI	p
Risk factor group												
Absent/Low	Reference	–	–	Reference	–	–	Reference	–	–	Reference	–	–
Intermediate	1.53	1.10–2.13	0.011	1.32	0.92–1.9	0.13	1.58	1.11–2.25	0.011	1.47	0.99–2.16	0.054
High	2.4	1.37–4.21	0.002	1.91	1.04–3.52	0.038	1.87	1.03–3.4	0.04	1.96	1.02–3.8	0.045

Model 1 unadjusted

Model 2 adjusted for age and treatment arm

*HR* hazard ratio, *CI* confidence intervals

As sensitivity analyses, we have compared patients with absent information for at least one of the variables included in the score (missing group) with the rest of the patients (non-missing group). Both groups have overlapping age distribution and treatment arm (Additional file 1: Table S3). The Kaplan Meier for the two outcomes is also completely comparable (Additional file 1: Figure S1).

Two additional sensitivity analyses were performed by using different categorization of risk factors. Results of the Cox models evaluating association with MACEs and OS confirm the associations of the risk factors with the two outcomes highlighted in the main analysis (Additional file 1: Table S4).

Table 4 shows the NNT values at median follow-up time-point and for both outcomes analysed. For this specific analysis, the comparison has been made towards the group with the highest number of risk factors and the hazard ratios are relative to this new reference.

**Table 4:** Number Needed to Treat (NNT) calculated using the hazard ratios (HR) and the survival probability in the high risk group at the time-point of 7.5 yr

	MACE				All-cause mortality			
	HR (95%CI)	Survival probability in high risk group	NNT (95%CI)	Patients still at risk (N)	HR (95%CI)	Survival probability in high risk group	NNT (95%CI)	Patients still at risk (N)
vs Absent/Low	0.5 (0.28–0.96)	0.29	4.3 (3.0–69.6)	151	0.5 (0.26–0.98)	0.47	4.7 (2.3–137.9)	197
vs intermediate	0.7 (0.39–1.22)	0.29	7.4 (3.7–13.9)	151	0.75 (0.40–0.98)	0.47	10.3 (3.1–137.9)	197

Considering the median follow-up, around 7.5 years for both outcomes, the NNT was 4.3 and 7.4 for the MACE outcome and 4.7 and 10.3 for the OS outcome, respectively towards the 0–1 risk factor and 2–3 risk-factor groups.

## Discussion

This post-hoc analysis demonstrates for the first time in a clinical trial which compared multifactorial versus standard intervention in type 2 diabetic population at high CV risk that CV prognosis and all-cause death significantly worsen with increased number of CV risk factors not reaching therapeutic goal. These results appear even more clinically interesting because they are achieved in a population undergoing primary CV prevention.

Although it is necessary to manage the CV disease burden in diabetes as effectively as possible, RCTs have not completely clarified the most effective overall therapeutic strategy. RCTs have almost always demonstrated the impact of a single CV risk factor and rarely have intensive multifactorial approaches been analysed.

A cohort study of about 270,000 type 2 diabetic patients were registered in the Swedish National Diabetes Register and matched with over 1,350,000 controls [14]. During a median follow-up of 5.7 years, subjects studied were assessed according to age categories as well as the presence of five CV risk factors (high glycated hemoglobin level, high LDL cholesterol level, albuminuria, smoking, and high blood pressure). A strict relationship between increasing number of CV risk factors not within target ranges and a higher risk of MACEs was observed. Particularly, younger diabetic subjects with multiple CV risk factors on target benefited most in terms of MACEs reduction. Notably, type 2 diabetic patients who had all five of the risk factor variables assessed within target ranges showed similar risks of death, MI, and stroke as compared with the general population. In contrast to the NID-2 study, in this very large Swedish cohort diabetic kidney disease (DKD) was either mild or absent in the majority of patients (mean estimated glomerular filtration rate (eGFR) 84 mL/min and less than 5% with macroalbuminuria). Moreover, achievement of targets for multiple risk factors was uncommon (5%), as was to be expected in an observational study. The findings from the Swedish Register are a call to action on the need for an intensive multifactorial therapeutic approach; however, according to the EUROASPIRE IV survey, a multi-drug approach to the main CV risk factors is not sufficient to achieve the goals suggested by scientific societies in the diabetic patient [15]. Hence, a multidisciplinary approach in real life and above all RCTs designed for this purpose are needed [16–20].

A meta-analysis of 7 RCTs did not support that intensive multifactorial intervention compared to standard of care reduced the risk of non-fatal MI, non-fatal stroke, CV disease mortality and overall mortality in microalbuminuric type 2 diabetic patients [21].

Intriguingly, the same meta-analysis showed intensive multifactorial risk factor control intervention significantly lowered blood pressure but showed a non-significant trend of reduction in HbA1c, total cholesterol, LDL cholesterol, triglyceride, and albuminuria.

Interestingly, these latter findings are partially supported by another meta-analysis of 19 RCTs in patients with type 2 diabetes. It was observed that multifactorial interventions significantly reduced the risk of non-fatal MI, but did not lower non-fatal stroke, CV disease and overall mortality [22].

Until last year, the Danish study Steno-2 and the Japanese study J-DOIT3 were the main studies which compared multifactorial intensive treatment and standard of care in type 2 diabetes [23, 24]. While the former only evaluated a microalbuminuric population, the latter analysed a diabetic population not selected for DKD. Steno-2 showed a significant reduction in MACEs and microvascular complications after an average of 7.8 years in the intensive treatment group, while the intervention group in J-DOIT3 did not reach a significant difference as compared to control group regarding fatal and non-fatal CV events after an average of 8 years. Furthermore, the post-intervention follow-up of the Steno-2 study observed a reduction in mortality in the intensive treatment arm [25].

Originally, the NID-2 trial obtained similar results as compared to Steno-2 that had an intervention phase lasting less than half (3.8 years).

But above all, these results were observed through a multicenter study on a larger population on primary prevention with patients equally distributed between the two genders, much closer to replicating the reality of type 2 diabetic patients regarding eGFR and with microvascular damage assessed by both albuminuria and DR [26]. Thus, the NID-2 study recruited a very high-risk CV population but on primary prevention, many years before recent ESC/EASD guidelines redefined the grading of CV risk in diabetes.

For decades, the guidelines of Scientific Societies have indicated HbA1c and blood glucose values as CV risk factors goals that patients with diabetes should achieve. Over the years these suggestions have often become more stringent as for the LDL cholesterol target, alongside the choice of both antihypertensive and antiplatelet drugs to use in the diabetic patients with high CV risk [3]. Assessed in their entirety, these goals are often not achieved both in the few RCTs with multifactorial treatment and in real life, both due to objective difficulty in achieving them and often because of therapeutic inertia [23, 24, 27]. Moreover, the target of single CV risk factor is often not reached in RCTs [28]. Therefore, the awareness that the achievement of a wide number of CV risk factors on target reduces CV morbidity and mortality may drive the physician and the patient to accept a multi-drug treatment, usually poorly tolerated in the daily management of diabetes. In the intensive multifactorial treatment arm of the NID-2 study the achievement of both a high percentage of subjects on target for a single risk factor and a high overall number of risk factors on target confirms that it is possible to achieve goals set out in guidelines in the clinical setting.

Our results about the association between risk factors and outcomes suggested that the risk factors might be a mediator of the intervention. Nevertheless, the association adjusted for intervention prevent the effect of other possible confounding related to itself.

This study has several strengths. Firstly, as far as we know, it is the first RCT evaluating intensive multifactorial treatment versus SoC which demonstrates that as the number of risk factors on target increases, the CV prognosis progressively improves. Secondly, these results may represent an important motivation for overcoming both the therapeutic inertia of doctors and the poor adherence of diabetics for multidrug therapy.

However, there are several limitations of the study. Firstly, post-hoc analysis results in an assessment beyond the original study design. In fact, the three analyzed groups are not homogeneous. In particular, the 4 CV risk factor group is smaller than the other two groups, this is due to the choice of variables to classify the risk factors groups.

Instead, the reason why the first two blocks were combined is to have larger groups and more robust estimates. On the other hand, this paper analyzed the same primary and secondary outcomes as in the NID-2 study, therefore the parameters analyzed are the same as in the original design of this RCT. Furthermore, despite the multicenter design of the study, the recommendation based on the study sample size was to distribute the subjects into three clusters according to the number of risk factors on target. Thus, this sub-division did not allow us to analyse the effect of achieving a single target at a time on the endpoints.

Moreover, only a minority of patients from MT group recorded data about physical activity and urinary sodium excretion as measure of dietary sodium intake. Therefore, the impact of non-pharmacological treatment on study outcomes was not analysed.

Finally, randomization by center and not by patient results in unblinded assignment, reducing both power and precision compared to an individually randomized study, and the ability to control for both known and unknown confounding variables. However, as described elsewhere, the main results of the study were adjusted for the cluster factor due to randomization. Moreover, the results were adjusted for the main variables resulted either unbalanced or clinically and statistically associated with outcome [4].

## Conclusions

A major challenge for all national health systems is the reduction of CV morbidity and mortality in individuals with high CV risk. According to recent international guidelines, future RCTs will have to evaluate how to achieve this important milestone in patients undergoing primary CV prevention. Unfortunately, the RCTs that have evaluated the application of the guidelines on the main risk factors are very few, discordant and sometimes with important limitations. This post-hoc analysis of the NID-2 study indicates that an increase in the number of risk factors at target correlates with reduction in MACEs and mortality in the very high CV risk diabetic population undergoing primary CV prevention. These findings represent a call to action for all clinicians involved in the management of such patients.

## Supplementary Information

**Additional file 1: Table S1**. Test of Likelihood ratio for interaction term. **Table S2**. Test of proportional-hazards assumption. **Table S3.** Sensitivity analysis comparing patients with absent information for at least one of the variables included in the score (missing group) and those without missing data. **Table S4.** Risks for MACEs and all-cause mortality by risk group at univariate Cox model and after adjustment for confounding variables. **Figure S1**. Sensitivity analysis comparing survival estimates for MACE and overall survival (OS) in patients with absent information for at least one of the variables included in the score (missing group) and those without missing data.

## Data Availability

All data relevant to the study are included in the article or uploaded as supplementary information.

## Abbreviations

CV: Cardiovascular
CI: Confidence Interval
DKD: Diabetic kidney disease
DR: Diabetic retinopathy
DBP: Diastolic blood pressure
eGFR: Estimated glomerular filtration rate
HbA1c: Glycated haemoglobin
HR: Hazard Ratio
LDL: Low density lipoprotein
MACEs: Major adverse cardiovascular events
MI: Myocardial infarction
MT: Multifactorial intensive therapy
NID-2: Nephropathy In Diabetes type 2
OS: Overall survival
RCTs: Randomized clinical trials
RAS: Renin-Angiotensin System
PTCA: Revascularization procedures
SoC: Standard-of-Care
SBP: Systolic blood pressure
T2DM: Type 2 diabetes mellitus

## References

[1] Emerging Risk Factors Collaboration, Sarwar N, Gao P, Seshasai SR et al. Diabetes mellitus, fasting blood glucose concentration, and risk of vascular disease: a collaborative meta-analysis of 102 prospective studies [published correction appears in Lancet. 2010 Sep 18;376(9745):958. Hillage, H L [corrected to Hillege, H L]]. Lancet. 2010;375(9733):2215–2222. 10.1016/S0140-6736(10)60484-9 10.1016/S0140-6736(10)60484-9 PMC2904878 20609967

[2] Stamler J, Vaccaro O, Neaton JD, Wentworth D (1993). Diabetes, other risk factors, and 12-yr cardiovascular mortality for men screened in the Multiple Risk Factor Intervention Trial. Diabetes Care.

[3] Cosentino F, Grant PJ, Aboyans V et al. ESC Scientific Document Group. 2019 ESC Guidelines on diabetes, pre-diabetes, and cardiovascular diseases developed in collaboration with the EASD. Eur Heart J. 2020 Jan 7;41(2):255–323. 10.1093/eurheartj/ehz486. Erratum in: Eur Heart J. 2020 Dec 1;41(45):4317. PMID: 31497854. 10.1093/eurheartj/ehz486 31497854

[4] Sasso FC, Pafundi PC, Simeon V et al. NID-2 Study Group Investigators. Efficacy and durability of multifactorial intervention on mortality and MACEs: a randomized clinical trial in type-2 diabetic kidney disease. Cardiovasc Diabetol. 2021;20(1):145. 10.1186/s12933-021-01343-1. PMID: 34271948; PMCID: PMC8285851. 10.1186/s12933-021-01343-1 PMC8285851 34271948

[5] American Diabetes Association. Standards of medical care in diabetes. Diabetes Care. 2005;28(Suppl 1): S4-S36. Erratum in: Diabetes Care. 2005 Apr;28(4):990. PMID: 15618112. 15618112

[6] Associazione Medici Diabetologi (AMD)—Società Italiana di Diabetologia (SID)—Standard italiani per la cura del diabete mellito 2005

[7] European Society of Hypertension-European Society of Cardiology Guidelines Committee. 2003 European Society of Hypertension-European Society of Cardiology guidelines for the management of arterial hypertension. J Hypertens. 2003;21(6): 1011–53. 10.1097/00004872-200306000-00001 10.1097/00004872-200306000-00001 12777938

[8] De Backer G, Ambrosioni E, Borch-Johnsen K (2003). Third Joint Task Force of European and Other Societies on Cardiovascular Disease Prevention in Clinical Practice. European guidelines on cardiovascular disease prevention in clinical practice. Third Joint Task Force of European and Other Societies on Cardiovascular Disease Prevention in Clinical Practice. Eur Heart J.

[9] Powers WJ, Rabinstein AA, Ackerson T et al. American Heart Association Stroke Council. 2018 Guidelines for the early management of patients with acute ischemic stroke: a guideline for healthcare professionals from the American Heart Association/American Stroke Association. Stroke. 2018;49(3):e46-e110. 10.1161/STR.0000000000000158. Epub 2018 Jan 24. Erratum in: Stroke. 2018;49(3):e138. Erratum in: Stroke. 2018; PMID: 29367334. 10.1161/STR.0000000000000158 29367334

[10] Ponikowski P, Voors AA, Anker SD et al. ESC Scientific Document Group. 2016 ESC Guidelines for the diagnosis and treatment of acute and chronic heart failure: the task force for the diagnosis and treatment of acute and chronic heart failure of the European Society of Cardiology (ESC) Developed with the special contribution of the Heart Failure Association (HFA) of the ESC. Eur Heart J. 2016;37(27):2129–2200. 10.1093/eurheartj/ehw128. Epub 2016 May 20. Erratum in: Eur Heart J. 2016; PMID: 27206819. 10.1093/eurheartj/ehw128 27206819

[11] Thygesen K, Alpert JS, Jaffe AS et al. ESC Scientific Document Group. Fourth universal definition of myocardial infarction (2018). Eur Heart J. 2019;40(3):237–269. 10.1093/eurheartj/ehy462. PMID: 30165617. 10.1093/eurheartj/ehy462 30165617

[12] Wilkinson CP, Ferris FL 3rd, Klein RE et al. Global Diabetic Retinopathy Project Group. Proposed international clinical diabetic retinopathy and diabetic macular edema disease severity scales. Ophthalmology. 2003;110(9):1677–82. 10.1016/S0161-6420(03)00475-5. 10.1016/S0161-6420(03)00475-5 13129861

[13] Altman DG, Andersen PK (1999). Calculating the number needed to treat for trials where the outcome is time to an event. BMJ.

[14] Rawshani A, Rawshani A, Franzén S (2018). Risk factors, mortality, and cardiovascular outcomes in patients with type 2 diabetes. N Engl J Med.

[15] Gyberg V, De Bacquer D, De Backer G et al.; EUROASPIRE Investigators. Patients with coronary artery disease and diabetes need improved management: a report from the EUROASPIRE IV survey: a registry from the EuroObservational Research Programme of the European Society of Cardiology. Cardiovasc Diabetol. 2015;14:133. 10.1186/s12933-015-0296-y. PMID: 26427624; PMCID: PMC4591740. 10.1186/s12933-015-0296-y PMC4591740 26427624

[16] Sasso FC, Marfella R, Pagano A (2015). Lack of effect of aspirin in primary CV prevention in type 2 diabetic patients with nephropathy: results from 8 years follow-up of NID-2 study. Acta Diabetol.

[17] Sasso FC, Chiodini P, Carbonara O et al. Nephropathy In Type 2 Diabetes Study Group. High cardiovascular risk in patients with Type 2 diabetic nephropathy: the predictive role of albuminuria and glomerular filtration rate. The NID-2 Prospective Cohort Study. Nephrol Dial Transplant. 2012;27(6):2269–74. 10.1093/ndt/gfr644. 10.1093/ndt/gfr644 22090446

[18] Sasso FC, Lascar N, Ascione A et al. NID-2 study group. Moderate-intensity statin therapy seems ineffective in primary cardiovascular prevention in patients with type 2 diabetes complicated by nephropathy. A multicenter prospective 8 years follow up study. Cardiovasc Diabetol. 2016;15(1):147. 10.1186/s12933-016-0463-9 10.1186/s12933-016-0463-9 PMC5062846 27733159

[19] Minutolo R, Sasso FC, Chiodini P (2006). Management of cardiovascular risk factors in advanced type 2 diabetic nephropathy: a comparative analysis in nephrology, diabetology and primary care settings. J Hypertens.

[20] Sasso FC, De Nicola L, Carbonara O (2006). Cardiovascular risk factors and disease management in type 2 diabetic patients with diabetic nephropathy. Diabetes Care.

[21] Usman M, Gillies CL, Khunti K, Davies MJ (2018). Effects of intensive interventions compared to standard care in people with type 2 diabetes and microalbuminuria on risk factors control and cardiovascular outcomes: a systematic review and meta-analysis of randomised controlled trials. Diabetes Res Clin Pract.

[22] Seidu S, Achana FA, Gray LJ, Davies MJ, Khunti K (2016). Effects of glucose-lowering and multifactorial interventions on cardiovascular and mortality outcomes: a meta-analysis of randomized control trials. Diabet Med.

[23] Gaede P, Vedel P, Larsen N, Jensen GV, Parving HH, Pedersen O (2003). Multifactorial intervention and cardiovascular disease in patients with type 2 diabetes. N Engl J Med.

[24] Ueki K, Sasako T, Okazaki Y et al. J-DOIT3 Study Group. Effect of an intensified multifactorial intervention on cardiovascular outcomes and mortality in type 2 diabetes (J-DOIT3): an open-label, randomised controlled trial. Lancet Diabetes Endocrinol. 2017;5(12):951–964. 10.1016/S2213-8587(17)30327-3. 10.1016/S2213-8587(17)30327-3 29079252

[25] Gaede P, Lund-Andersen H, Parving HH, Pedersen O (2008). Effect of a multifactorial intervention on mortality in type 2 diabetes. N Engl J Med.

[26] Sasso FC, Pafundi PC, Gelso A (2019). Telemedicine for screening diabetic retinopathy: the NO BLIND Italian multicenter study. Diabetes Metab Res Rev.

[27] Jiang He, Zhengbao Zhu, Joshua D. Bundy, Kirsten S. Dorans, Jing Chen, L. Lee Hamm. Trends in cardiovascular risk factors in US adults by race and ethnicity and socioeconomic status, 1999–2018. JAMA. 2021; 326(13): 1286–1298. 10.1001/jama.2021.15187 10.1001/jama.2021.15187 PMC8493438 34609450

[28] Monami M, Candido R, Pintaudi B, Targher G, Mannucci E; of the SID-AMD joint panel for Italian Guidelines on Treatment of Type 2 Diabetes. Improvement of glycemic control in type 2 diabetes: a systematic review and meta-analysis of randomized controlled trials. Nutr Metab Cardiovasc Dis. 2021;31(9):2539–2546. 10.1016/j.numecd.2021.05.010. 10.1016/j.numecd.2021.05.010 34158243

