# Supplementary material for: The number of risk factors not at target is associated with cardiovascular risk in a type 2 diabetic population with albuminuria in primary cardiovascular prevention. Post-hoc analysis of the NID-2 trial
Source: Cardiovasc Diabetol. 2022 Nov 7;21:235. doi: 10.1186/s12933-022-01674-7 (PMC9641842; doi:10.1186/s12933-022-01674-7)
Supplement: Supplementary file 1 — Additional file 1: Table S1. Test of Likelihood ratio for interaction term. Table S2. Test of proportional-hazards assumption. Table S3. Sensitivity analysis comparing patients with absent information for at least one of the variables included in the score (missing group) and those without missing data. Table S4. Risks for MACEs and all-cause mortality by risk group at univariate Cox model and after adjustment for confounding variables. Figure S1. Sensitivity analysis comparing survival estimates for MACE and overall survival (OS) in patients with absent information for at least one of the variables included in the score (missing group) and those without missing data. [file 12933_2022_1674_MOESM1_ESM.docx]

**SUPPLEMENTARY MATERIAL FOR THE PAPER**

**The number of risk factors not at target is associated with cardiovascular risk in a type 2 diabetic population with albuminuria in primary cardiovascular prevention. Post-hoc analysis of the NID-2 trial**

Ferdinando Carlo Sasso,^a^* PhD, Vittorio Simeon,^b^* PhD, Raffaele Galiero,^a^* MD, Alfredo Caturano, ^a^ MD, Luca De Nicola,^a^ PhD, Paolo Chiodini,^b^ MSc, Luca Rinaldi,^a^ PhD, Teresa Salvatore,^c^ MD, Miriam Lettieri,^d^ PhD, Riccardo Nevola,^a^ PhD, Celestino Sardu,^a^ PhD, Giovanni Docimo,^a^ MD, Giuseppe Loffredo,^a^ D, Raffaele Marfella,^a^ PhD, Luigi Elio Adinolfi,^a^ MD, Roberto Minutolo,^a^ PhD; on behalf of NID-2 study group Investigators

^a^ University of Campania “Luigi Vanvitelli”, Department of Advanced Medical and Surgical Sciences, Piazza Luigi Miraglia 2, I-80138 – Naples, Italy

^b^ University of Campania “Luigi Vanvitelli”, Medical Statistics Unit, Department of Physical and Mental Health and Preventive Medicine, Piazza Luigi Miraglia 2, I-80138 – Naples, Italy

^c^ University of Campania “Luigi Vanvitelli”, Department of Precision Medicine, Via De Crecchio 7, I-80138 – Naples, Italy

^d^ Division of Cardiovascular Sciences, Faculty of Biology, Medicine and Health, The University of Manchester, 3.31 Core Technology Facility, 46 Grafton Street, Manchester M13 9NT, UK;

**Supplementary Table 1**. Test of Likelihood ratio for interaction term.

| MACE | Log likelihood | Likelihood-ratio test |
| --- | --- | --- |
| Complete model | -756.63 | LR chi2 (2) = 0.13; p=0.94 |
| Plus interaction term | -756.56 |  |
| OS |  |  |
| Complete model | -673.33 | LR chi2 (2) = 1.21; p=0.27 |
| Plus interaction term | -672.72 |  |

Complete model: Risk factor groups + Age + Treatment arm; Interaction term: Risk factor groups*Treatment arm. The assumption for likelihood ratio test is that complete model is nested in model with interaction

**Supplementary Table 2**. Test of proportional-hazards assumption

| MACE | Variable | rho | Chi2 | df | p |
| --- | --- | --- | --- | --- | --- |
|  | Risk factor |  |  |  |  |
|  | Absent/Low | - | - | 1 | - |
|  | Intermediate | -0.03 | 0.14 | 1 | 0.71 |
|  | High | -0.07 | 0.75 | 1 | 0.38 |
|  | Treatment | 0.05 | 0.41 | 1 | 0.52 |
|  | Age | -0.07 | 1.00 | 1 | 0.32 |
|  | GLOBAL |  | 1.88 | 4 | 0.76 |
| Overall Survival | Risk factor |  |  |  |  |
|  | Absent/Low | - | - | 1 | - |
|  | Intermediate | -0.11 | 1.37 | 1 | 0.24 |
|  | High | -0.14 | 2.66 | 1 | 0.11 |
|  | Treatment | 0.06 | 0.39 | 1 | 0.53 |
|  | Age | -0.04 | 0.29 | 1 | 0.59 |
|  | GLOBAL |  | 3.20 | 4 | 0.52 |

Phtest was performed in STATA using Schoenfeld residuals

**Supplementary Table 3**: Sensitivity analysis comparing patients with absent information for at least one of the variables included in the score (missing group) and those without missing data.

|  | Non-Missing | Missing | p |
| --- | --- | --- | --- |
| N | 317 | 51 |  |
| Age (years) | 70.3 (9.3) | 70.7 (8.0) | 0.8 |
| Treatment arm |  |  |  |
| MT | 169 (53.3%) | 30 (58.8%) | 0.46 |
| SoC | 148 (46.7%) | 21 (41.2%) |  |

Age years are mean±SD. T-test and Chi-square test were used to compare Non-Missing and Missing group. Missing group is represented by 51 patients (30 to MT and 21 to SoC arm) with absent information for at least one of the variables included in the score.

**Supplementary Table 4.** Risks for MACEs and all-cause mortality by risk group at univariate Cox model and after adjustment for confounding variables.

|  | MACEs | | | | | | |  | All-cause mortality | | | | | | |  |
| --- | --- | --- | --- | --- | --- | --- | --- | --- | --- | --- | --- | --- | --- | --- | --- | --- |
|  |  | Regrouping 1 |  |  |  | Regrouping 2 |  |  |  | Regrouping 1 |  |  |  | Regrouping 2 |  | |
|  | HR | 95%CI | p |  | HR | 95%CI | p |  | HR | 95%CI | p |  | HR | 95%CI | p | |
| Risk factor |  |  |  |  |  |  |  |  |  |  |  |  |  |  |  | |
| Absent/Low | Ref. | - | - |  | Ref. | - | - |  | Ref. | - | - |  | Ref. | - | - | |
| Intermediate | 1.49 | 1.03 - 2.17 | 0.033 |  | 1.29 | 0.91 - 1.85 | 0.15 |  | 1.49 | 1.01 - 2.23 | 0.046 |  | 1.33 | 0.92 - 1.94 | 0.13 | |
| High | 1.83 | 1.24 - 2.72 | 0.003 |  | 2.14 | 1.24 - 3.7 | 0.006 |  | 1.80 | 1.18 - 2.72 | 0.006 |  | 1.66 | 0.92 - 2.9 | 0.089 | |

HR, hazard ratio; CI confidence intervals.

Regrouping 1: Score grouped as 0-1, 2 and 3-4 (Absent/Low n=166, Intermediate n=82, High n=69)

Regrouping 2: Combining high SBP and/or DBP in one risk factor with subsequent regrouping in 0-1, 2, 3 (Absent /Low n=207, Intermediate n=87, High n=23)

Supplementary Figure 1. Sensitivity analysis comparing survival estimates for MACE and overall survival (OS) in patients with absent information for at least one of the variables included in the score (missing group) and those without missing data.

| **MACE** | **OS** |
| --- | --- |
| **** | **** |
